# Tuberculosis knowledge, misconceptions/myths in adults: findings from Lesotho, Malawi, Namibia and Zambia Demographic Health Surveys (2013–2016)

**DOI:** 10.1186/s13104-018-3884-6

**Published:** 2018-10-31

**Authors:** Godfrey Musuka, Vonai Teveredzi, Farirai Mutenherwa, Innocent Chingombe, Munyaradzi Mapingure

**Affiliations:** 1 ICAP at Columbia University, Harare, Zimbabwe; 2 Ark Foundation, Harare, Zimbabwe; 3 Biomedical Research and Training Institute, Harare, Zimbabwe

**Keywords:** Tuberculosis, HIV, Community, Knowledge, Attitudes, Lesotho, Malawi, Myths, Namibia, Zambia, Demographic and Health Surveys

## Abstract

**Objective:**

To determine TB knowledge and misconceptions/myths amongst HIV positive and negative adults using Demographic Health Survey data from Lesotho, Malawi, Namibia and Zambia.

**Results:**

Overall 97% (n = 58,107) of both male and female respondents irrespective of their HIV status had heard of tuberculosis out of whom 82.6% knew that it can be cured. Knowledge that TB is spread in air when coughing or sneezing was 73.8%. Significantly higher proportions of HIV positive men and women than their HIV negative counterparts, had ever heard about TB, knew that it is transmitted through air when coughing and sneezing and also that it can be cured. However interestingly, significantly higher proportions of HIV positive men and women, than their HIV negative counterparts, had the misconception that TB is spread through sharing utensils or would overall say they did not know how it is spread. TB knowledge was significantly higher among individuals who are less than 26 years of age compared to those who were older.

## Introduction

Tuberculosis (TB) is a global public health challenge despite a notable decline in the incidence and mortality rates worldwide [1]. In 2016, 10, 4 million cases of TB were recorded worldwide. Africa bears the highest global TB/HIV burden. Of the 1.4 million new cases of TB reported in 2016, amongst people who were HIV-positive, 74% were in Africa [2]. A significant proportion of those infected with TB may not be aware of their status or get a late diagnosis while those who initiate treatment seldom complete the course [3]. The combined effect of late treatment and non-adherence to treatment severely undermine efforts to curb the TB disease burden. This calls for increased efforts to understand the causal factors. TB remains the leading cause of death among all infectious diseases with a case fatality ratio of 16% [1]. Approximately 1.3 million TB deaths were recorded in 2016 among HIV negative people, with an additional 374,000 deaths recorded among HIV-positive people, globally. Out of the four countries discussed in this paper, Lesotho had the highest TB mortality rate (HIV + TB only) estimated to be 238 per 100,000 population followed by Mozambique, Zambia and Namibia at 114, 74 and 35 per 100,000 population respectively [1]. In the absence of early diagnosis and treatment, the combined effect of HIV and TB drastically shorten human life expectancy.

On the one hand HIV accelerates the progression from latent to active TB and to development of TB disease while on the other hand HIV weakens the immune system, increasing the risk of TB in people with HIV. Evidence has shown that the risk of developing TB is estimated to be 16–27 times greater in HIV positive people compared to those without the infection [4].

Key interventions for TB control have focused intensified case finding of smear positive TB among patients presenting with cough and early initiation of treatment HIV testing and counselling, early initiation on ART, isoniazid preventive treatment in people living with HIV, [5, 6]. Early initiation on ART in TB and HIV co-infected patients on TB treatment improves uptake and continuation on ART [7]. Approximately 53 million deaths were averted by TB treatment between 2000 and 2016, with 17% deaths averted from HIV positive people on ART. A total of 11 million deaths were averted in Africa due to TB control [1]. The direct observed treatment short (DOTS) is one of the best means of increasing adherence among TB infected patients on treatment. However, it relies on passive presentation of patients hence its success or failure depends on the prevailing perceptions of the community about TB, stigma, awareness of early signs as well as access to health services. These factors may influence individuals and community level health seeking behavior.

In this paper we analyzed data from Demographic Health Surveys from four countries in Southern Africa to understand knowledge and myths/perceptions of TB among HIV infected people. The analysis provides baseline data on evaluating whether reducing myths/misconceptions stigma and increasing TB knowledge in the community improves early diagnosis and patient outcomes among HIV positive people.

## Main text

### Methods

#### Study area and data sources

The study area was Lesotho, Malawi, Namibia and Zambia, four countries in southern Africa with very high TB notification rates.^1^ This study used secondary data from the most recent demographic and health surveys conducted in the participating countries during the period 2013–2016.

#### Participant sampling

Study participants were enrolled into the surveys using a two-stage sampling procedure to identify eligible households and participants [8]. This study uses data from a total of 2526 enumeration areas (EAs). Data from 57,697 survey participants aged 15–54 years was used in this analysis. Data was from 30,906 women and 26,791 men across the four countries. In all the study countries anonymous HIV testing was implemented with informed consent obtained for every sampled individual. Additionally, consent was obtained from a parent or guardian on behalf of any participants under the age of 16. HIV screening and confirmatory testing was conducted using the appropriate national HIV testing algorithm of each study country [8].

#### Statistical analysis

We used STATA Version 15.1, Texas USA to conduct our statistical analysis. Variables used were age, gender, highest level of education attained, marital status, religion, type of residence (urban/rural), wealth index, and exposure to mass media (frequency of reading newspaper/magazine, listening to radio, and watching television).

We first present the demographic characteristics of the combined participants from the four countries using simple proportions. We also conducted Chi square test to determine the association between TB knowledge, myths/misconceptions and HIV positivity split by gender. There after we calculated adjusted and non-adjusted odds ratios for determinants of TB misconceptions.

### Results

#### Sample description

The combined sample used in this analysis was 58,107 out of which 42% were aged between 15 and 24 years, see Table 1 below. The majority (62%) lived in rural area. Close to half had attained either primary or secondary education, 45 and 42% respectively. Half of the women were married while the combined weighted HIV prevalence was 14% for the four southern Africa countries.

**Table 1:** Weighted baseline characteristics of the combined DHS sample from Lesotho 2014, Malawi 2015, Namibia 2013 and Zambia 2014

Variable	Frequency n (%) N = 58,107
Age group in years	
15–19	12,989 (23)
20–24	10,474 (19)
25–29	8682 (15)
30–34	7710 (14)
35–39	6563 (11)
40–44	5014 (9)
45–49	3747 (6)
50–54	1637 (2)
55–59	954 (1)
60–64	337 (0)
Gender	
Female	31,451 (53)
Male	26,656 (47)
Type of residence	
Urban	22,637 (38)
Rural	35,470 (62)
Highest education level	
None	4185 (7)
Primary	26,233 (45)
Secondary	24,399 (42)
Higher	3272 (6)
Marital status	
Never in Union	21,453 (37)
Married	29,406 (51)
Living with partner	2255 (4)
Widowed	1475 (2)
Divorced	2003 (3)
Separated	1515 (3)
Religion	
No religion	574 (1)
Other	2897 (6)
Other christian	8984 (17)
Pentecostal	1315 (3)
Protestant or anglican or methodist	24,344 (47)
Roman catholic	13,902 (27)
HIV status	
Negative	50,269 (86)
Positive	7838 (14)

#### Knowledge of TB and misconceptions among participants

Overall 97% (n = 58,107) of both male and female respondents irrespective of their HIV status had heard of tuberculosis out of which 82.6% knew that it can be cured, see Table 2 below.

**Table 2:** Percentage of adult men and women from Lesotho, Malawi, Namibia and Zambia responses to TB knowledge related questions by HIV status

Variable	Female HIV+	Female HIV−	P-value	Male HIV+	Male HIV−	P-value	Overall
Ever hear of TB	97.6	96.4	< 0.001	98.5	97.3	< 0.001	97.0
TB spread by air when coughing or sneezing	78.8	70.7	<0.001	81.4	75.2	<0.001	73.8
TB spread through sharing utensils	20.4	15.7	< 0.001	24.5	18.7	< 0.001	17.8
TB spread through touching a person with TB	3.5	3.1	0.253	4.0	3.7	0.407	3.4
TB spread through sharing food	3.9	3.8	0.735	5.4	4.9	0.258	4.3
TB spread through sexual contact	3.5	4.0	0.210	3.5	3.4	0.831	3.8
TB spread through mosquito bite	0.1	0.3	0.104	0.2	0.2	0.612	0.2
TB spread through other means	4.0	2.7	< 0.001	7.4	7.2	0.737	4.9
Don’t Know how TB is spread	14.4	21.7	< 0.001	5.9	8.4	< 0.001	14.7
TB can be cured	90.2	79.4	< 0.001	91.8	83.3	< 0.001	82.6
Would keep it a secret if a family member get TB	38.8	40.1	0.269	31.3	31.8	0.620	36.1

Knowledge that TB is spread in air when coughing or sneezing was 73.8%. There were varying levels of misconception about modes of TB transmission ranging from a mere 0.2% who said it is spread through mosquito bite to a high of 17.8% who said it can be spread through sharing utensils.

Significantly higher proportions of HIV positive men and women had ever heard about TB, knew that it is transmitted through air when coughing and sneezing and also that it can be cured. However interestingly, significantly higher proportions of HIV positive men and women had the misconception that TB is spread through sharing utensils or would overall say they did not know how it is spread.

We calculated unadjusted and adjusted odd ratios and their 95% confidence interval for determinate of correct knowledge of TB without misconception, see Table 3. We found significantly correct knowledge for those individuals who are less than 26 years compared to those who are above 40 years, females compared to males those with basic education compared to those without, the married compared to those who were never in union, the Pentecostal compared to people of no religion, those who read newspapers or magazines at least once a week compared to those who don’t, those who listen to the radio at least once a week or almost every day compared to those who don’t, those belonging to the rich wealth quintiles compared to the poorest.

**Table 3:** Determinants of correct knowledge about tuberculosis transmission among participants by univariate and multivariable analyses

Variable	Unadjusted OR (95% CI)	P-value	Adjusted OR (95% CI)	P-value
Age				
> 40	1		1	
> 25–40	1.10 (1.04–1.16)	< 0.001	1.04 (0.98–1.11)	0.186
≤ 25	1.16 (1.09–1.22)	< 0.001	1.08 (1.00–1.16)	0.045
Gender				
Female	1			
Male	1.11 (1.08–0.16)	< 0.001	1.19 (1.14–1.25)	< 0.001
Residence				
Urban	1			
Rural	0.86 (0.82–0.89)	< 0.001	1.23 (1.16–1.31)	< 0.001
Highest education level				
None	1			
Primary	1.31 (1.21–1.42)	< 0.001	1.26 (1.15–1.37)	< 0.001
Secondary	1.86 (1.72–2.02)	< 0.001	1.74 (1.58–1.92)	< 0.001
Higher	2.43 (2.16–2.73)	< 0.001	2.03 (1.77–2.34)	< 0.001
Marital status				
Never in Union	1			
Married	0.96 (0.92–1.00)	0.059	1.08 (1.02–1.15)	0.01
Living with partner	0.90 (0.81–1.00)	0.055	0.96 (0.83–1.09)	0.505
Widowed	1.01 (0.88–1.16)	0.908	1.12 (0.96–1.31)	0.151
Divorced	0.88 (0.79–0.99)	0.031	1.06 (0.93–1.20)	0.406
Separated	0.90 (0.79–1.02)	0.102	0.99 (0.85–1.14)	0.851
Religion				
No religion	1			
Other	0.90 (0.71–1.13)	0.354	0.72 (0.56–0.91)	0.006
Other christian	1.02 (0.82–1.27)	0.877	0.79 (0.63–0.99)	0.043
Pentecostal	1.77 (1.37–2.27)	0.000	1.30 (1.00–1.69)	0.049
Protestant or anglican or methodist	0.76 (0.61–0.94)	0.010	0.58 (0.46–0.73)	< 0.000
Roman catholic	0.94 (0.75–1.16)	0.553	0.71 (0.57–0.89)	0.003
Frequency of reading newspaper or magazine				
Not at all	1			
Less than once a week	1.27 (1.20–1.34)	< 0.001	1.05 (0.99–1.12)	0.096
At least once a week	1.34 (1.26–1.42)	< 0.001	1.08 (1.00–1.15)	0.042
Almost everyday	1.28 (1.17–1.41)	< 0.001	1.10 (0.99–1.22)	0.078
Frequency of listening to the radio				
Not at all	1			
Less than once a week	1.10 (1.04–1.17)	< 0.001	1.04 (0.97–1.11)	0.278
At least once a week	1.38 (1.31–1.45)	< 0.001	1.19 (1.12–1.26)	< 0.000
Almost everyday	1.18 (1.11–1.25)	< 0.001	1.15 (1.07–1.23)	< 0.000
Frequency of watching television				
Not at all	1			
Less than once a week	1.18 (1.11–1.25)	< 0.001	1.04 (0.97–1.12)	0.258
At least once a week	1.41 (1.33–1.50)	< 0.001	1.05 (0.98–1.14)	0.178
Almost everyday	1.19 (1.12–1.27)	< 0.001	0.91 (0.83–0.99)	0.033
SES				
Poorest	1			
Poorer	1.27 (1.19–1.35)	< 0.001	1.20 (1.12–1.29)	< 0.001
Middle	1.38 (1.29–1.47)	< 0.001	1.30 (1.21–1.39)	< 0.001
Richer	1.53 (1.43–1.63)	< 0.001	1.43 (1.32–1.54)	< 0.001
Richest	2.00 (1.87–2.13)	< 0.001	1.74 (1.58–1.91)	< 0.001

### Discussion

Researchers have increasingly focused on the factors that impact on prevention and treatment of HIV and TB because of the interplay between the two. Most studies conducted in Africa and Asia have focused on disease-specific knowledge and stigma [3, 9, 10]. Both HIV and TB cause severe illness and are transmissible diseases, hence are highly stigmatized. TB stigma has been found to be one of the social determinants that negatively affect the success of the tuberculosis control and treatment interventions [11]. One of the negative effects of stigma is that it may lead to isolation and discrimination and instills fear to seek appropriate medical attention. Knowledge of facts on TB could therefore be an essential determinant of health-seeking behavior and adherence to both prevention and treatment efforts.

The current study findings show that communities in the four SADC countries are highly accommodative of TB patients. This high acceptance of people with TB has been demonstrated across the various socio-demographic characteristics of the respondents such as age, sex, highest levels of education attained and religious affiliation. However, our study also showed the existence of myths which may fuel stigmatization of the affected individuals as well as negatively affect the implementation and ultimately, the success of national TB programmes.

Patients with good knowledge of TB are likely to seek treatment early and adhere to treatment thus reducing ongoing transmission in the community. All the programs need to develop information for dissemination to help patients understand the facts on about TB transmission and control and reduce the risk of development of tuberculosis drug resistance and delayed diagnosis/treatment leading to enhanced disease transmissions and increased severity respectively.

Southern African Development Community tuberculosis programmes should enhance investments in qualitative studies, inorder to isolate the key drivers of myths and misconceptions in our communities and individuals at large and distil these findings into appropriate actionable policy level interventions.

## Limitations

Demographic Health Survey are cross-sectional studies, which do not provide the causal relationships between variables analyzed. Additionally, by design, the surveys only interview women age 15–49 years and men age 15–54 years and adolescence less than 15 who might also have inadequate knowledge and or myths/misconceptions are not included in the individuals who responded to the questionnaire.

## Abbreviations

EA: enumeration areas
ELISA: enzyme-linked immunosorbent assay
HIV: human immunosuppression virus
DHS: Demographic Health Survey
SADC: Southern African Development Community
TB: tuberculosis

## Footnotes

1 World TB report, 2017.
